# 4-Chloro-6-meth­oxy­pyrimidin-2-amine

**DOI:** 10.1107/S160053681204528X

**Published:** 2012-11-10

**Authors:** Kaliyaperumal Thanigaimani, Nuridayanti Che Khalib, Suhana Arshad, Ibrahim Abdul Razak

**Affiliations:** a School of Physics, Universiti Sains Malaysia, 11800 USM, Penang, Malaysia

## Abstract

The title compound, C_5_H_6_ClN_3_O, is essentially planar with a maximum deviation of 0.0256 (11) Å for all non-H atoms. In the crystal, adjacent mol­ecules are linked by a pair of N—H⋯N hydrogen bonds, forming an inversion dimer with an *R*_2_^2^(8) ring motif. The dimers are further linked *via* N—H⋯O hydrogen bonds into an undulating sheet structure parallel to the *bc* plane.

## Related literature

For the biological activity of pyrimidine and amino­pyrimidine derivatives, see: Hunt *et al.* (1980 ▶); Baker & Santi (1965 ▶). For related structures, see: Schwalbe & Williams (1982 ▶); Hu *et al.* (2002 ▶); Chinnakali *et al.* (1999 ▶); Skovsgaard & Bond (2009 ▶). For hydrogen-bond motifs, see: Bernstein *et al.* (1995 ▶). For bond-length data, see: Allen *et al.* (1987 ▶). For stability of the temperature controller used for the data collection, see: Cosier & Glazer (1986 ▶).
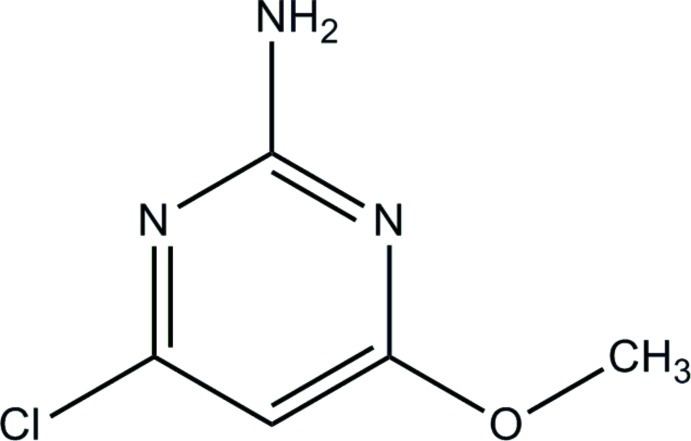


## Experimental

### 

#### Crystal data

C_5_H_6_ClN_3_O
*M* *_r_* = 159.58
Monoclinic, 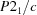

*a* = 3.7683 (2) Å
*b* = 16.4455 (2) Å
*c* = 10.7867 (2) Å
β = 94.550 (1)°
*V* = 666.36 (4) Å^3^
*Z* = 4
Mo *K*α radiation
μ = 0.50 mm^−1^
*T* = 100 K
0.49 × 0.28 × 0.21 mm

#### Data collection

Bruker SMART APEXII CCD area-detector diffractometer
Absorption correction: multi-scan (*SADABS*; Bruker, 2009 ▶) *T*_min_ = 0.791, *T*_max_ = 0.904
9524 measured reflections
2436 independent reflections
2266 reflections with *I* > 2σ(*I*)
*R*_int_ = 0.016

#### Refinement

*R*[*F* ^2^ > 2σ(*F* ^2^)] = 0.027
*wR*(*F* ^2^) = 0.070
*S* = 1.06
2436 reflections
100 parameters
H atoms treated by a mixture of independent and constrained refinement
Δρ_max_ = 0.67 e Å^−3^
Δρ_min_ = −0.26 e Å^−3^

### 

Data collection: *APEX2* (Bruker, 2009 ▶); cell refinement: *SAINT* (Bruker, 2009 ▶); data reduction: *SAINT*; program(s) used to solve structure: *SHELXTL* (Sheldrick, 2008 ▶); program(s) used to refine structure: *SHELXTL*; molecular graphics: *SHELXTL*; software used to prepare material for publication: *SHELXTL* and *PLATON* (Spek, 2009 ▶).

## Supplementary Material

Crystal structure: contains datablock(s) global, I. DOI: 10.1107/S160053681204528X/is5214sup1.cif

Structure factors: contains datablock(s) I. DOI: 10.1107/S160053681204528X/is5214Isup2.hkl

Supplementary material file. DOI: 10.1107/S160053681204528X/is5214Isup3.cml

Additional supplementary materials:  crystallographic information; 3D view; checkCIF report

## Figures and Tables

**Table 1:** Hydrogen-bond geometry (Å, °)

*D*—H⋯*A*	*D*—H	H⋯*A*	*D*⋯*A*	*D*—H⋯*A*
N3—H2*N*3⋯O1^i^	0.828 (16)	2.251 (17)	3.0699 (11)	170.1 (15)
N3—H1*N*3⋯N1^ii^	0.850 (16)	2.183 (16)	3.0335 (12)	180 (2)

Symmetry codes: (i) 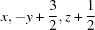; (ii) 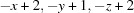.

**Fig. 1.**
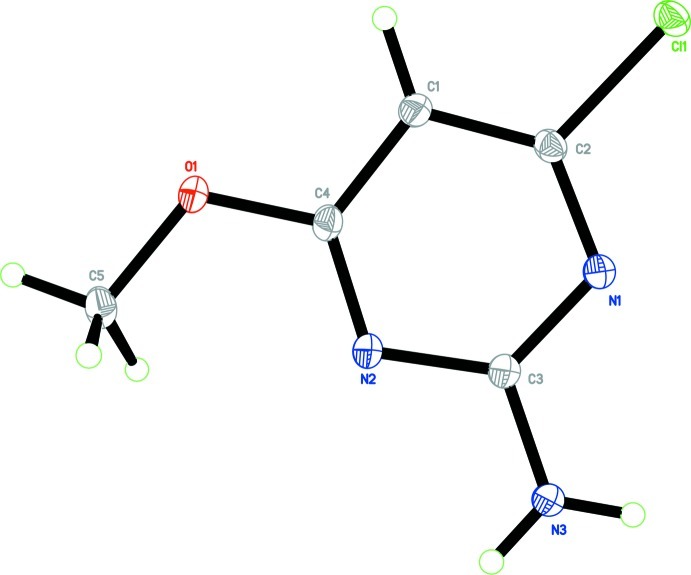
The molecular structure of the title compound with atom labels with 50% probability displacement ellipsoids.

**Fig. 2.**
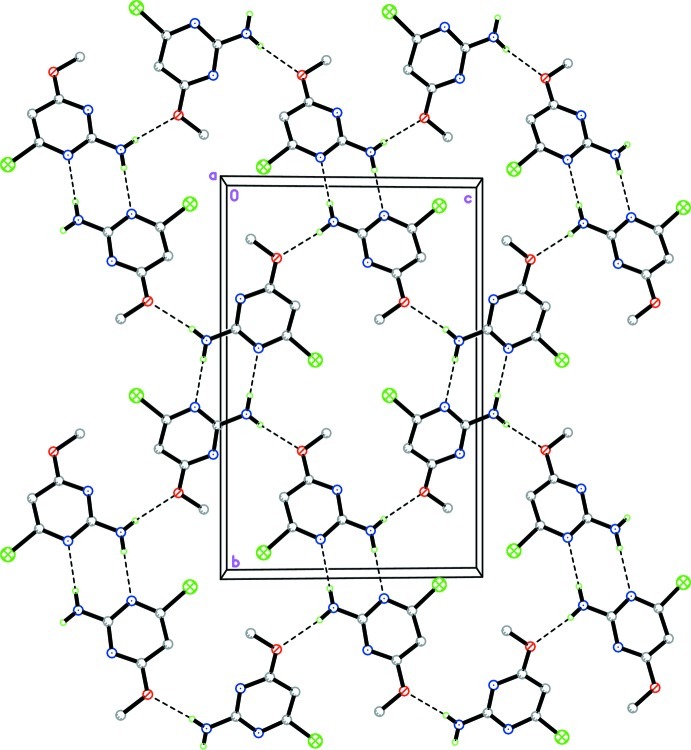
The crystal packing of the title compound. The H atoms not involved in the intermolecular interactions (dashed lines) have been omitted for clarity.

## supplementary crystallographic information

### Comment

Pyrimidine and aminopyrimidine derivatives are biologically important compounds as they occur in nature as components of nucleic acids. Some aminopyrimidine derivatives are used as antifolate drugs (Hunt *et al.*, 1980; Baker & Santi, 1965). The crystal structures of aminopyrimidine derivatives (Schwalbe & Williams, 1982), aminopyrimidine carboxylates (Hu *et al.*, 2002) and co-crystal structures (Chinnakali *et al.*, 1999; Skovsgaard & Bond, 2009) have been reported. In order to study some interesting hydrogen bonding interactions, the synthesis and structure of the title compound, (I), is presented here.

The title compound (Fig. 1) is essentially planar, with atom C5 deviating a maximum of 0.0256 (11) Å from a mean plane of non-H atoms. The bond lengths (Allen *et al.*, 1987) and angles are normal. In the crystal structure (Fig. 2), molecules are linked by a pair of N3—H1N3···N1^ii^ hydrogen bonds (symmetry code in Table 1) into an inversion dimer, forming an *R*_2_^2^(8) (Bernstein *et al.*, 1995) ring motif. These molecules are self-assembled *via* N3—H2N3···O1^i^ hydrogen bonds (graph-set notation C(6); symmetry code in Table 1), which interconnect the dimers resulting in a wavy sheet parallel to the *bc* plane.

### Experimental

A hot ethanol solutions (20 ml) of 2-amino-4-chloro-6-methoxypyrimidine (36 mg, Aldrich) was warmed over a heating magnetic stirrer hotplate for a few minutes. The resulting solution was allowed to cool slowly at room temperature. Single crystals of the title compound (I) appeared from the mother liquor after a few days.

### Refinement

N-bound H atoms were located in a difference Fourier maps and refined freely [N—H = 0.828 (16) and 0.850 (16) Å]. The remaining H atoms were positioned geometrically (C–H = 0.95–0.98 Å) and were refined using a riding model, with *U*_iso_(H) = 1.2*U*_eq_(C) or 1.5*U*_eq_(methyl C). A rotating group model was used for the methyl group. Two outliers were omitted (1 8 14 and 0 1 2) in the final refinement.

### Crystal data

**Table:**

C_5_H_6_ClN_3_O	*F*(000) = 328
*M**_r_* = 159.58	*D*_x_ = 1.591 Mg m^−^^3^
Monoclinic, *P*2_1_/*c*	Mo *K*α radiation, λ = 0.71073 Å
Hall symbol: -P 2ybc	Cell parameters from 6060 reflections
*a* = 3.7683 (2) Å	θ = 3.8–32.6°
*b* = 16.4455 (2) Å	µ = 0.50 mm^−^^1^
*c* = 10.7867 (2) Å	*T* = 100 K
β = 94.550 (1)°	Block, colourless
*V* = 666.36 (4) Å^3^	0.49 × 0.28 × 0.21 mm
*Z* = 4	

### Data collection

**Table:**

Bruker SMART APEXII CCD area-detector diffractometer	2436 independent reflections
Radiation source: fine-focus sealed tube	2266 reflections with *I* > 2σ(*I*)
Graphite monochromator	*R*_int_ = 0.016
φ and ω scans	θ_max_ = 32.6°, θ_min_ = 2.3°
Absorption correction: multi-scan (*SADABS*; Bruker, 2009)	*h* = −5→5
*T*_min_ = 0.791, *T*_max_ = 0.904	*k* = −24→21
9524 measured reflections	*l* = −16→14

### Refinement

**Table:**

Refinement on *F*^2^	Primary atom site location: structure-invariant direct methods
Least-squares matrix: full	Secondary atom site location: difference Fourier map
*R*[*F*^2^ > 2σ(*F*^2^)] = 0.027	Hydrogen site location: inferred from neighbouring sites
*wR*(*F*^2^) = 0.070	H atoms treated by a mixture of independent and constrained refinement
*S* = 1.06	*w* = 1/[σ^2^(*F*_o_^2^) + (0.0306*P*)^2^ + 0.3355*P*] where *P* = (*F*_o_^2^ + 2*F*_c_^2^)/3
2436 reflections	(Δ/σ)_max_ = 0.001
100 parameters	Δρ_max_ = 0.67 e Å^−^^3^
0 restraints	Δρ_min_ = −0.26 e Å^−^^3^

### Special details

**Table:**

Experimental. The crystal was placed in the cold stream of an Oxford Cryosystems Cobra open-flow nitrogen cryostat (Cosier & Glazer, 1986) operating at 100.0 (1) K.
Geometry. All e.s.d.'s (except the e.s.d. in the dihedral angle between two l.s. planes) are estimated using the full covariance matrix. The cell e.s.d.'s are taken into account individually in the estimation of e.s.d.'s in distances, angles and torsion angles; correlations between e.s.d.'s in cell parameters are only used when they are defined by crystal symmetry. An approximate (isotropic) treatment of cell e.s.d.'s is used for estimating e.s.d.'s involving l.s. planes.
Refinement. Refinement of *F*^2^ against ALL reflections. The weighted *R*-factor *wR* and goodness of fit *S* are based on *F*^2^, conventional *R*-factors *R* are based on *F*, with *F* set to zero for negative *F*^2^. The threshold expression of *F*^2^ > σ(*F*^2^) is used only for calculating *R*-factors(gt) *etc*. and is not relevant to the choice of reflections for refinement. *R*-factors based on *F*^2^ are statistically about twice as large as those based on *F*, and *R*- factors based on ALL data will be even larger.

### Fractional atomic coordinates and isotropic or equivalent isotropic displacement parameters (Å^2^)

**Table:**

	*x*	*y*	*z*	*U*_iso_*/*U*_eq_	
Cl1	1.11362 (6)	0.546310 (13)	0.64838 (2)	0.01425 (6)	
O1	0.57563 (19)	0.80520 (4)	0.79576 (6)	0.01449 (13)	
N1	0.9448 (2)	0.57688 (5)	0.87272 (7)	0.01299 (14)	
N2	0.6906 (2)	0.70257 (5)	0.93883 (7)	0.01284 (14)	
N3	0.8057 (3)	0.59594 (5)	1.07440 (8)	0.01996 (17)	
C1	0.8338 (2)	0.68624 (5)	0.72638 (8)	0.01284 (15)	
H1A	0.8437	0.7075	0.6448	0.015*	
C2	0.9490 (2)	0.60954 (5)	0.76040 (8)	0.01137 (14)	
C3	0.8137 (2)	0.62570 (5)	0.95924 (8)	0.01305 (15)	
C4	0.7000 (2)	0.73004 (5)	0.82415 (8)	0.01155 (15)	
C5	0.4336 (3)	0.85141 (6)	0.89378 (9)	0.01568 (16)	
H5A	0.3073	0.8992	0.8583	0.024*	
H5B	0.6286	0.8691	0.9532	0.024*	
H5C	0.2680	0.8174	0.9366	0.024*	
H2N3	0.722 (4)	0.6245 (10)	1.1281 (15)	0.027 (4)*	
H1N3	0.876 (4)	0.5475 (10)	1.0891 (15)	0.023 (4)*	

### Atomic displacement parameters (Å^2^)

**Table:**

	*U*^11^	*U*^22^	*U*^33^	*U*^12^	*U*^13^	*U*^23^
Cl1	0.01773 (10)	0.01199 (10)	0.01346 (10)	0.00039 (7)	0.00388 (7)	−0.00276 (7)
O1	0.0206 (3)	0.0093 (3)	0.0136 (3)	0.0025 (2)	0.0019 (2)	0.0013 (2)
N1	0.0169 (3)	0.0103 (3)	0.0119 (3)	0.0017 (2)	0.0022 (2)	0.0000 (2)
N2	0.0170 (3)	0.0092 (3)	0.0124 (3)	0.0018 (2)	0.0017 (2)	0.0004 (2)
N3	0.0359 (5)	0.0129 (4)	0.0119 (3)	0.0084 (3)	0.0069 (3)	0.0022 (3)
C1	0.0171 (4)	0.0105 (3)	0.0110 (3)	0.0002 (3)	0.0014 (3)	0.0003 (3)
C2	0.0128 (3)	0.0099 (3)	0.0116 (3)	−0.0006 (3)	0.0018 (3)	−0.0018 (3)
C3	0.0171 (4)	0.0103 (3)	0.0118 (3)	0.0014 (3)	0.0020 (3)	0.0005 (3)
C4	0.0130 (3)	0.0086 (3)	0.0130 (3)	−0.0001 (3)	0.0004 (3)	0.0003 (3)
C5	0.0176 (4)	0.0115 (4)	0.0182 (4)	0.0029 (3)	0.0026 (3)	−0.0013 (3)

### Geometric parameters (Å, º)

**Table:**

Cl1—C2	1.7449 (9)	N3—H2N3	0.828 (16)
O1—C4	1.3485 (10)	N3—H1N3	0.850 (16)
O1—C5	1.4393 (11)	C1—C2	1.3743 (12)
N1—C2	1.3266 (11)	C1—C4	1.4035 (12)
N1—C3	1.3539 (11)	C1—H1A	0.9500
N2—C4	1.3201 (11)	C5—H5A	0.9800
N2—C3	1.3584 (11)	C5—H5B	0.9800
N3—C3	1.3378 (12)	C5—H5C	0.9800
			
C4—O1—C5	117.38 (7)	N3—C3—N1	117.35 (8)
C2—N1—C3	114.89 (8)	N3—C3—N2	117.28 (8)
C4—N2—C3	115.86 (8)	N1—C3—N2	125.37 (8)
C3—N3—H2N3	118.7 (11)	N2—C4—O1	119.46 (8)
C3—N3—H1N3	119.1 (11)	N2—C4—C1	124.50 (8)
H2N3—N3—H1N3	122.1 (15)	O1—C4—C1	116.04 (8)
C2—C1—C4	113.28 (8)	O1—C5—H5A	109.5
C2—C1—H1A	123.4	O1—C5—H5B	109.5
C4—C1—H1A	123.4	H5A—C5—H5B	109.5
N1—C2—C1	126.09 (8)	O1—C5—H5C	109.5
N1—C2—Cl1	114.95 (6)	H5A—C5—H5C	109.5
C1—C2—Cl1	118.96 (7)	H5B—C5—H5C	109.5
			
C3—N1—C2—C1	−0.18 (13)	C4—N2—C3—N1	0.61 (14)
C3—N1—C2—Cl1	−179.36 (6)	C3—N2—C4—O1	178.63 (8)
C4—C1—C2—N1	−0.63 (13)	C3—N2—C4—C1	−1.55 (13)
C4—C1—C2—Cl1	178.52 (6)	C5—O1—C4—N2	−0.73 (12)
C2—N1—C3—N3	−179.55 (9)	C5—O1—C4—C1	179.44 (8)
C2—N1—C3—N2	0.22 (13)	C2—C1—C4—N2	1.55 (13)
C4—N2—C3—N3	−179.62 (9)	C2—C1—C4—O1	−178.62 (8)

### Hydrogen-bond geometry (Å, º)

**Table:**

*D*—H···*A*	*D*—H	H···*A*	*D*···*A*	*D*—H···*A*
N3—H2*N*3···O1^i^	0.828 (16)	2.251 (17)	3.0699 (11)	170.1 (15)
N3—H1*N*3···N1^ii^	0.850 (16)	2.183 (16)	3.0335 (12)	180 (2)

Symmetry codes: (i) *x*, −*y*+3/2, *z*+1/2; (ii) −*x*+2, −*y*+1, −*z*+2.
